# A Green Platform for Preparation of the Well-Defined Polyacrylonitrile: ^60^Co γ-ray Irradiation-Initiated RAFT Polymerization at Room Temperature

**DOI:** 10.3390/polym9010026

**Published:** 2017-01-17

**Authors:** Shuangshuang Zhang, Lu Yin, Junzhi Wang, Wei Zhang, Lifen Zhang, Xiulin Zhu

**Affiliations:** 1 Suzhou Key Laboratory of Macromolecular Design and Precision Synthesis, Jiangsu Key Laboratory of Advanced Functional Polymer Design and Application, State and Local Joint Engineering Laboratory for Novel Functional Polymeric Materials, College of Chemistry, Chemical Engineering and Materials Science, Soochow University, Suzhou 215123, China; sgny19910327@163.com (S.Z.); yinlu0701@163.com (L.Y.); j624wang@uwaterloo.ca (J.W.); zhanglifen@suda.edu.cn (L.Z.); 2 Faculty of Engineering, University of Waterloo, Waterloo, ON N2L 3G1, Canada

**Keywords:** acrylonitrile, controlled polymerization, reversible addition–fragmentation chain transfer (RAFT) polymerization, γ-ray irradiation

## Abstract

^60^Co γ-ray irradiation-initiated reversible addition–fragmentation chain transfer (RAFT) polymerization at room temperature with 2-cyanoprop-2-yl 1-dithionaphthalate (CPDN) as the chain transfer agent was first applied to acrylonitrile (AN) polymerization, providing a “green” platform for preparing polyacrylonitrile (PAN)-based carbon fibers using an environment-friendly energy source. Various effects of dose rate, molar ratio of the monomer to the chain transfer agent, monomer concentration and reaction time on the AN polymerization behaviors were performed to improve the controllability of molecular the weight and molecular weight distribution of the obtained PAN. The feature of the controlled polymerization was proven by the first-order kinetics, linear increase of the molecular weight with the monomer conversion and a successful chain-extension experiment. The molecular weight and molecular weight distribution of PAN were characterized by size exclusion chromatography (SEC). ^1^H NMR and Matrix assisted laser desorption ionization/time of flight mass spectra (MALDI-TOF-MS) confirmed the chain-end functionality of PAN, which also was supported by the successful chain-extension experiments of original PANs with acrylonitrile and styrene as the second monomers respectively.

## 1. Introduction

Because of its remarkable and unique performance, including rigidity, tensile strength and chemical resistance, polyacrylonitrile (PAN) as the precursor for carbon fibers plays an important role in many industries, such as aerospace, automotive, sports goods and so on [1]. It is well known that PAN with high molecular weight, narrow molecular weight distribution and high stereotacticity is necessary for preparing high-quality carbon fibers to gain various high-quality industrial products [2,3]. Nevertheless, controlling polymerization of acrylonitrile (AN) is still challenging for researchers because of its high reactivity and poor solubility of PAN, which may prevent improvement of the mechanical performance of PAN-based carbon fibers [4,5].

Shindo et al. first reported the successful synthesis of PAN-based carbon fibers in 1961 [6]. After that, several strategies such as bulk, solution or suspension polymerization and so on [7,8], have been attempted to prepare PAN precursors. However, side reactions and relatively strict polymerization conditions are still unavoidable factors that hinder the improvement of the mechanical performance of these materials [9]. In the 1990s, Nakano and Kunio reported the synthesis of well-defined PAN via anionic polymerization [10,11], which provided a good starting point for the structure characterization of PAN. Then, “living”/controlled radical polymerization (LRP) methods, including atom transfer radical polymerization (ATRP) [2,3,12], reversible addition–fragmentation chain transfer (RAFT) [13,14,15,16,17] polymerization, and single electron transfer-living radical polymerization (SET-LRP) [18,19,20], were investigated in detail to prepare PANs with devisable molecular weights, narrow molecular weight distributions and well-defined architectures.

Compared with other LRP techniques, RAFT polymerization is an outstanding strategy with high functional-group tolerance and without any metal catalyst. Up to now, there have been only some successful examples of RAFT polymerization of acrylonitrile using cumyl dithiobenzoate (CDB) [13], 2-cyanoprop-2-yl dithiobenzoate (CPDB) [14,15] or dibenzyl trithiocarbonate (DBTC) [16] as chain transfer agents (CTAs) by means of thermal initiation. Recently, at a lower temperature (30 °C), Moskowitz et al. prepared PAN with high molecular weight and narrow distribution using 2-cyano-2-propyl dodecyl trithiocarbonate (CPDT) as a RAFT agent, providing a new direction to our train of thought [21].

**Scheme 1:**
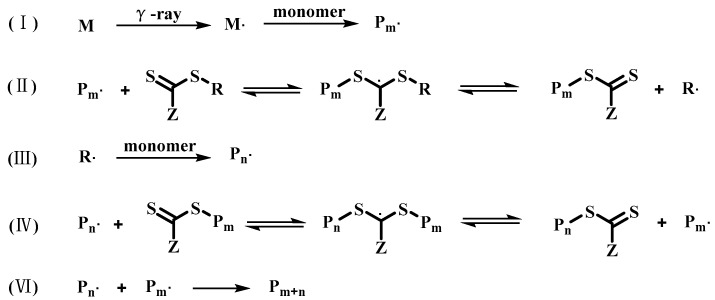
Possible mechanism for γ-ray irradiation-initiated reversible addition–fragmentation chain transfer (RAFT) polymerization.

Without using any thermal initiator or photoinitiator, ^60^Co γ-ray irradiation, as a kind of environment-friendly energy source, can be used to initiate vinyl polymerization, and to prepare graft or cross-linked copolymers to modify the properties of polymers [22,23,24,25]. This polymerization is often difficult to control and the obtained polymers have uncontrolled molecular weights and broad molecular weight distributions [26]. Nevertheless, Pan and coworkers, combining ^60^Co γ-ray irradiation and RAFT polymerization, first reported the “living”/controlled radical polymerization (LRP) of styrene (St), methyl acrylate (MA) and methyl methacrylate (MMA) respectively, in the presence of dibenzyl trithiocarbonate (DBTTC) in 2001 [27], which paved the way for irradiation-mediated “living”/controlled polymerization. After that, the same group systematically investigated irradiation-mediated RAFT polymerization of different vinyl monomers using different RAFT agents, such as DBTC [28,29], CTAs with *N*-groups [30], CTAs with *O*-groups [31], novel cyclic dixanthate [32] and so on. With respect to the polymerization process, Davis et al. reported that this γ-ray irradiation-mediated LRP would undergo a RAFT mechanism [33,34,35,36,37,38], as shown in Scheme 1.

**Scheme 2:**
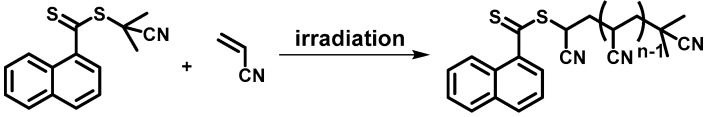
Synthetic route for RAFT polymerization of acrylonitrile (AN).

In this work, we attempted to apply ^60^Co γ-ray irradiation to the RAFT polymerization of acrylonitrile using commercial available 2-cyanoprop-2-yl 1-dithionaphthalate (CPDN) as the chain transfer agent at room temperature (Scheme 2). The various investigations including dose rate, molar ratio of the monomer to the chain transfer agent, monomer concentration and reaction time were performed to improve the controllability of the molecular weight and molecular weight distribution. This work will provide a new “green” platform for preparing the well-defined PANs via an environment-friendly energy source.

## 2. Experiment

### 2.1. Materials

Acrylonitrile (AN, Sinopharm Chemical Reagent, CP) and styrene (St, Sinopharm Chemical Reagent, Shanghai, China, 99%) were respectively purified by short Al_2_O_3_ columns before use. The 2-cyanoprop-2-yl 1-dithionaphthalate (CPDN) was synthesized according to literature procedures [39] and purified via column chromatography. Other reagents were purchased from Sinopharm Chemical Reagent Co., Ltd. and were used without any further purification.

### 2.2. Characterizations

The number-average molecular weight (*M*_n_) and molecular weight distribution (*M*_w_/*M*_n_) of the polymer were determined using TOSOH TSKgel SuperHM-M size exclusion chromatography (SEC, TOSOH, Tokyo, Japan) with a refractive-index detector equipped with an autosampler and calibrated with polystyrene standards. *N,N*-Dimethyl formamide (DMF) was used for the eluent at a flow rate of 0.65 mL/min and 40 °C. ^1^H NMR spectra of the polymers were recorded using a Bruker nuclear magnetic resonance instrument (300 MHz, Brucker, Kalsruhe, Germany) with tetramethylsilane (TMS) as the internal standard and DMSO-*d*_6_ as the solvent at room temperature. Matrix assisted laser desorption ionization/time of flight mass spectra (MALDI-TOF-MS) were acquired on an UltrafleXtreme MALDI-TOF mass spectrometer (Brucker, Kalsruhe, Germany) with a 1 kHz smart beam-II laser. The compound *trans*-2-[3-(4-*tert*-butyl-phenyl)-2-metyl-2-propenylidene]-malononitrile (DCTB, Aldrich, Beijing, China, >98%) as the matrix prepared in CDCl_3_ (20 mg/mL) and the cationizing agent sodium trifluoroacetate prepared in ethanol (10 mg/mL) were mixed in a ratio of 10/1 (*v*/*v*).

### 2.3. General Procedures for ^60^Co γ-ray Irradiation-Mediated RAFT Polymerization of AN

A typical solution polymerization procedure of AN is listed as follows. A 5 mL ampoule filled with CPDN, AN and EC was degassed by nitrogen-bubbling methods and polymerized under ^60^Co γ-ray irradiation. After the predetermined time, the mixture was dissolved in DMF and precipitated in an excess of methanol. After filtration, the polymer was dried in vacuum at 30 °C for 24 h.

### 2.4. Chain Extension of AN Using PAN as Macro-RAFT Agent

An amount of 15.26 mg PAN (sample in Table 1 (1c)), 0.41 g AN and 1.32 g EC were added into a 5 mL ampoule, degassed by nitrogen-bubbling methods and polymerized under ^60^Co γ-ray irradiation for 4 h. The mixture was dissolved in 2 mL DMF and precipitated in an excess of methanol. After filtration, PAN was dried in vacuum at 30 °C for 24 h (123 mg).

### 2.5. Chain Extension of PS Using PAN as Macro-RAFT Agent

An amount of 9.60 mg PAN (sample in Table 1 (1c)), 0.50 g St and 1.32 g EC were added into a 5 mL ampoule, degassed by nitrogen-bubbling methods and polymerized under ^60^Co γ-ray irradiation for 7 h. The mixture was dissolved in 0.5 mL DMF and precipitated in an excess of methanol. After filtration, PAN-*b*-PS was dried in vacuum at 30 °C for 24 h (47.5 mg).

## 3. Results and Discussion

### 3.1. General Procedures for ^60^Co γ-ray Irradiation-Mediated RAFT Polymerization of AN

**Figure 1:**
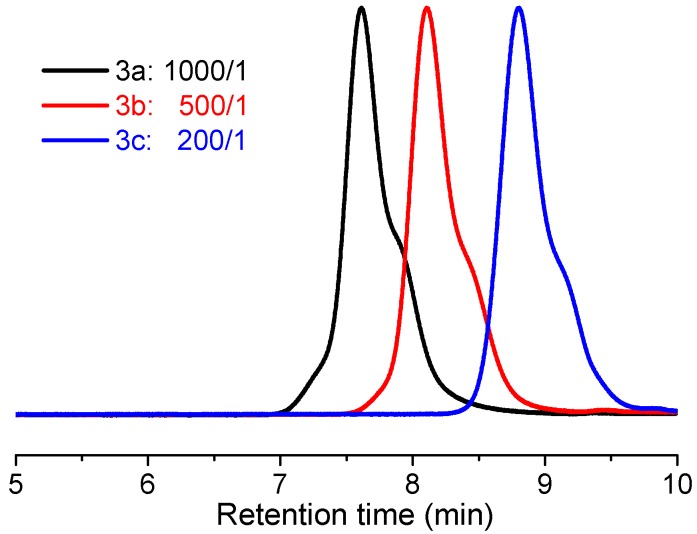
SEC chromatograms of 3a, 3b, 3c (Table 1). DMF was used as the eluent and PS as the calibration standard.

The route and possible mechanism of γ-ray irradiation-initiated RAFT polymerization of AN is shown in Scheme 1 and Scheme 2. Different from traditional RAFT polymerization, the initiating radical in γ-ray irradiation-initiated RAFT polymerization is generated by a monomer rather than an initiator [30]. In the current system, compared with DMF and DMSO, ethylene carbonate (EC) was employed as the solvent because of its inertia toward chain-transfer reaction in the RAFT polymerization process of AN [15]. First, the effects of dose rate, monomer concentration and molar ratio of the monomer to the chain transfer agent on polymerization behavior were investigated and the results were summarized in Table 1. As shown in Table 1 (1a–1c, 2a–2c or 3a–3c), we could find that the rate of polymerization, the molecular weight and molecular weight distribution (*M*_w_/*M*_n_) of the obtained PAN decreased with the decrease of [AN]_0_/[CPDN]_0_ molar ratio under the constant conditions of dose rate, monomer concentration and reaction time, indicating that these polymerizations developed towards a more controlled direction. Figure 1 showed SEC chromatograms of 3a, 3b and 3c (Table 1) with DMF as the eluent and PS as the calibration standard. From the SEC curves (Figure 1 and Figures S1 and S2), we could find that a little uplifted peak existed in the low molecular weight field, perhaps caused by the refrain from stirring in the polymerization process. In addition, with the decrease of dose rate under other constant conditions (Table 1 (1a, 2a, 3a)), the molecular weight of the obtained polymer also decreased. Meanwhile, molecular weight distribution (*M*_w_/*M*_n_) decreased with the decrease of the polymerization rate. Comparing 1b and 4a (Table 1), with the increase of monomer concentration, the polymerization became much more controllable. Actually, on account of poor solubility of PAN under high monomer concentration, some visible pale pink solid precipitated out from the solution during the polymerization process and the controllability of polymerization became much weaker under the condition of [AN]_0_/[CPDN]_0_ = 1000/1 (Figure S3).

### 3.2. Kinetics of AN Polymerization

**Figure 2:**
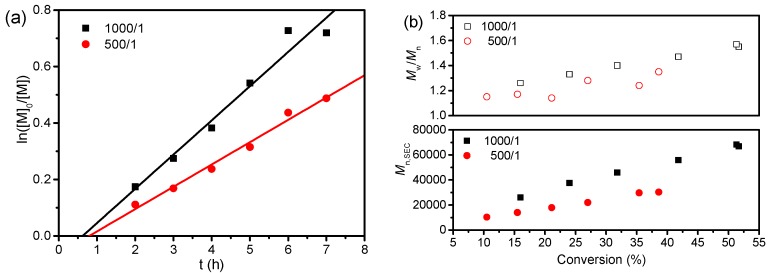
Kinetic plots of ln([M]_0_/[M]) as a function of time (**a**); evolution of the number-average molecular weight (*M*_n,SEC_) and molecular weight distribution (*M*_w_/*M*_n_) versus monomer conversions (**b**) for γ-ray irradiation-initiated RAFT polymerization of AN at 1.8 kGy/h with different [AN]_0_/[CPDN]_0_ ratios (1000/1 and 500/1).

According to the RAFT polymerization mechanism, the [M]_0_/[CTA]_0_ molar ratio has significant influence on the ^60^Co γ-ray irradiation-initiated RAFT polymerization of AN. A proper concentration of monomer or chain transfer agent is desirable and indispensable to gain proper molecular weights and narrow molecular weight distributions (*M*_w_/*M*_n_) of polymers [15,16]. Figure 2a showed the first-order kinetics of ln([M]_0_/[M]) as a function of polymerization time for ^60^Co γ-ray irradiation-initiated RAFT polymerization of AN at 1.8 kGy/h under the constant initial concentration of AN. These profiles of plots were approximately linear, indicating the stability of the number of the active centers during the polymerization process. The polymerization rate decreased with the decrease of the [AN]_0_/[CPDN]_0_ molar ratio. It could be observed that there was an inhibition period at the beginning of polymerization, perhaps because of the slow fragmentation of the intermediate radical (Scheme 1II) [33,34,35,36,37,38]. Figure 2b showed the dependence of the number-average molecular weight (*M*_n,SEC_) and molecular weight distribution (*M*_w_/*M*_n_) on the monomer conversions at different [AN]_0_/[CPDN]_0_ ratios. The *M*_n,GPC_ value of PAN increased linearly with the monomer conversion and the molecular weight distribution indices of PAN were kept under 1.4 in most cases. Furthermore, the molecular weight distribution decreased with the decrease of the [AN]_0_/[CPDN]_0_ molar ratios as a whole. All the results indicated that an appropriate increase of the CPDN molar concentration could make the polymerization behavior of AN become much more controllable.

**Figure 3:**
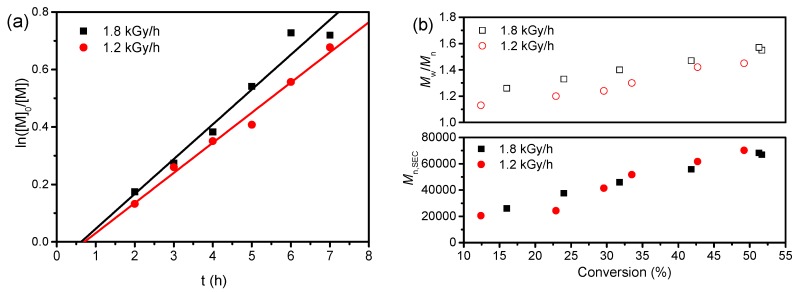
Kinetic plots of ln([M]_0_/[M]) as a function of time (**a**); evolution of the number-average molecular weight (*M*_n,SEC_) and molecular weight distribution (*M*_w_/*M*_n_) versus monomer conversions (**b**) for γ-ray irradiation-initiated RAFT polymerization with different dose rates ([AN]_0_/[CPDN]_0_ = 1000/1).

The effects of different dose rates on the RAFT polymerization of AN were also investigated. As presented in Figure 3a, the first-order kinetics of ln([M]_0_/[M]) as a function of time for ^60^Co γ-ray irradiation-initiated RAFT polymerization of AN with different dose rates (1.8 and 1.2 kGy/h) under the molar ratio of [AN]_0_/[CPDN]_0_ = 1000/1, were observed. The linear relationship between ln([M]_0_/[M]) and polymerization time meant that the number of active centers remains constant during the polymerization process. As expected, the rate of polymerization decreased in the order of 1.8 kGy/h > 1.2 kGy/h, indicating the relatively lower radical concentrations induced by ^60^Co γ-ray irradiation with the lower dose rate. There still existed an inhibition period at the beginning of polymerization. Figure 3b presented the evolution of the number-average molecular weight (*M*_n,SEC_) and molecular weight distribution (*M*_w_/*M*_n_) versus the monomer conversion under the molar ratio of [AN]_0_/[CPDN]_0_ = 1000/1. It could be seen that the *M*_n,SEC_ value of PAN increased linearly with monomer conversion and that molecular weight distribution indices of PAN were also kept under 1.4 in most cases. Meanwhile, molecular weight distribution indices broadened with the increase in dose rate as a whole, demonstrating that an appropriate decrease of the dose rate could make AN polymerize in a more controlled fashion.

### 3.3. Chain-End Functionality

**Figure 4:**
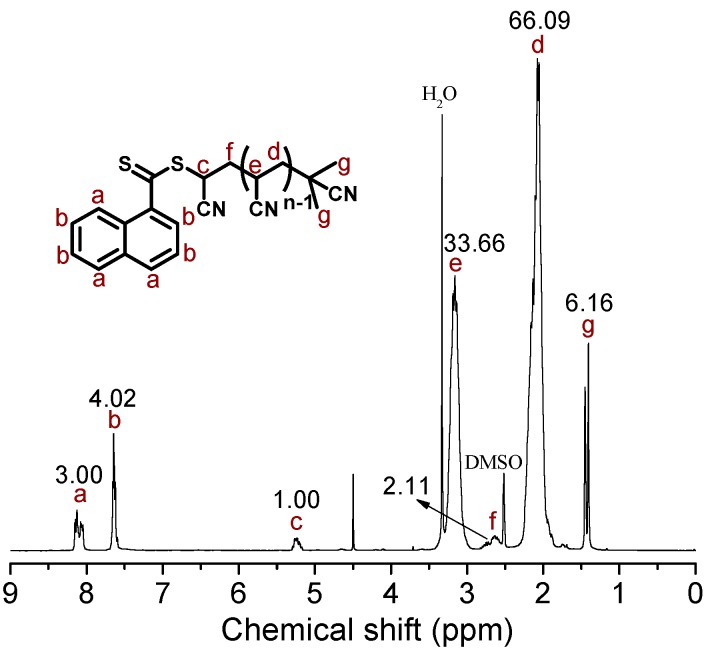
^1^H NMR spectrum of PAN with CPDN as the CTA in EC under γ-ray irradiation (1.8 kGy/h; *t* = 5 h; [AN] = 5.09 mol/L; [AN]_0_/[CPDN]_0_ = 200/1; Conv.% = 16.0%).

**Figure 5:**
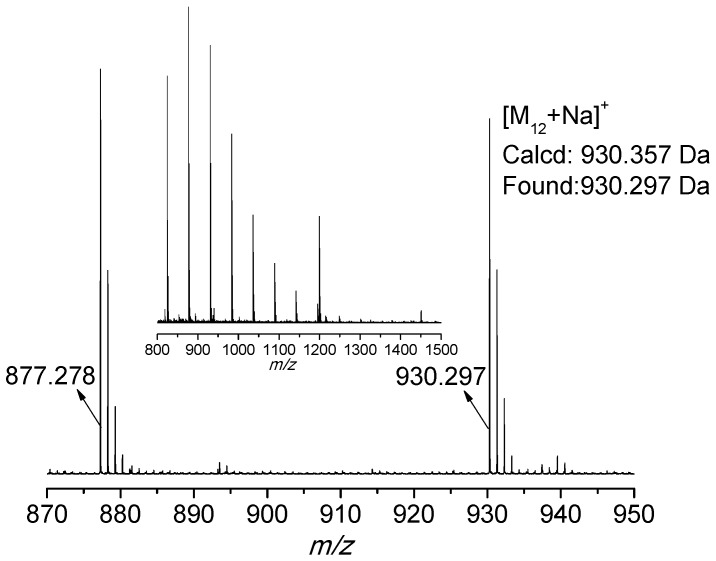
Expanded MALDI TOF mass spectra of PAN (0.6 kGy/h; *t* = 5 h; [AN] = 5.09 mol/L; [AN]_0_/[CPDN]_0_ = 200/1; Conv.% = 4.9%).

**Figure 6:**
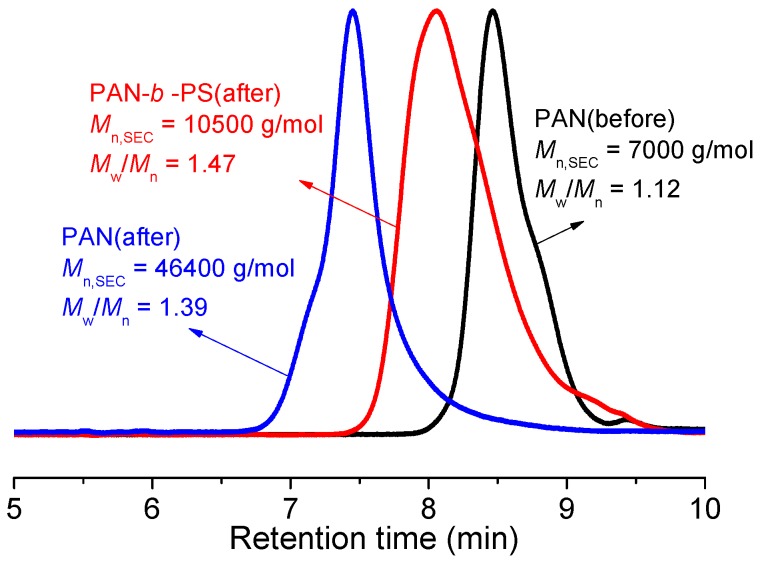
SEC chromatograms of PAN (before chain-extension), PAN-*b*-PS (after chain-extension) and PAN (after chain-extension). DMF was used as the eluent and PS standard as the calibration.

**Figure 7:**
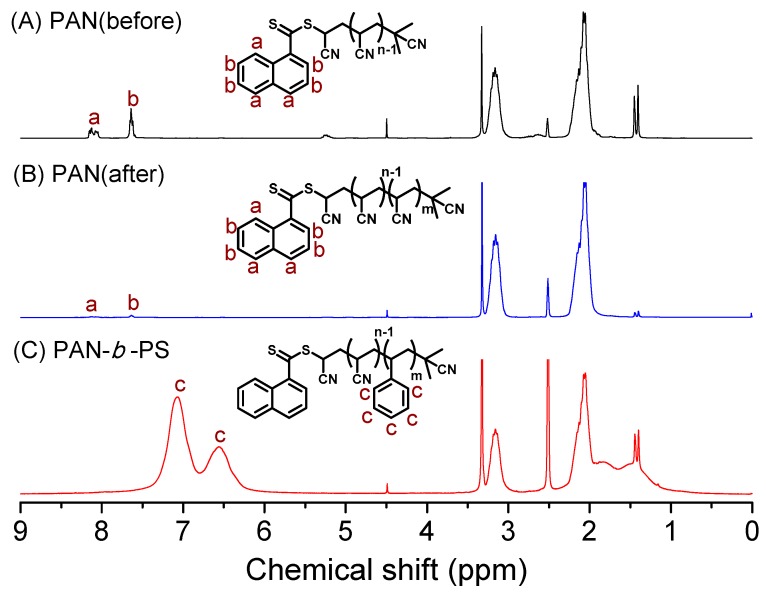
^1^H NMR spectrum of PAN (before chain-extension, **A**), PAN (after chain-extension, **B**) and PAN-*b*-PS (after chain-extension, **C**).

Efficient chain-end functionality is an important aspect of “living”/controlled radical polymerization. We characterized the chain-end functionality using ^1^H NMR (Figure 4) and MALDI-TOF-MS (Figure 5). Meanwhile, the chain-extension experiments with AN and St as the monomer using the obtained PAN as the macro-CTA were also performed. Figure 4 showed the ^1^H NMR spectrum of PAN prepared with CPDN as the CTA in EC under ^60^Co γ-ray irradiation. The aromatic proton signals (7.50–8.20 ppm) and signals of CH_3_ (1.20–1.50 ppm) in the CPDN unit were clearly observed. The (*a* + *b*)/*c*/*f*/*g* integration ratio was close to 7/1/2/6, confirming the efficient chain-end functionality of PAN. Furthermore, the molecular weight of PAN could be calculated by comparing the integration ratio value of aromatic protons from end groups with CH_2_ protons from the polymer main chain. The number-average molecular weight (*M*_n,NMR_) of PAN was near 2100 g/mol calculated by the ^1^H NMR spectrum corresponding to the *M*_n,th_ of 2000 g/mol. In Figure 5, the representative experimental peak value (*m*/*z*, 930.297 Da), corresponding to 12 repeat units of PAN with a sodium cation, is consistent with the theoretically calculated value (*m*/*z*, 930.357 Da). The difference of two adjacent peaks is 53.02 Da, equal to the mass of one acrylonitrile unit. The successful chain-extension experiments (Figure 6) showed the obvious peak shifts from the original PAN (*M*_n,SEC_ = 7000 g/mol, *M*_w_/*M*_n_ = 1.12) to polymers (PAN or PAN-*b*-PS), strongly proving the living feature of the previous polymerization. In the NMR spectra of copolymers (Figure 7), we also observed the characteristic peaks of PAN and PS respectively, further confirming the living feature of previous polymerization. Therefore, the above data confirmed the efficient chain-end functionality of PAN gained by ^60^Co γ-ray irradiation-mediated “living”/controlled polymerization.

## 4. Conclusions

^60^Co γ-ray irradiation-initiated reversible addition–fragmentation chain transfer (RAFT) polymerization at room temperature with 2-cyanoprop-2-yl 1-dithionaphthalate (CPDN) as the chain transfer agent was first applied to acrylonitrile (AN) polymerization. Various effects of dose rate, molar ratio of the monomer to the chain transfer agent, monomer concentration and reaction time on the AN polymerization behaviors were performed to improve the controllability of the molecular weight and molecular weight distribution of the obtained PAN. The feature of the controlled polymerization was proven by the first-order kinetics, linear increase of the molecular weight with the monomer conversion and a successful chain-extension experiment. This work provides a new “green” platform for preparing PAN-based carbon fibers using an environment-friendly energy source and benefits the optimization of the mechanical properties of PAN-based carbon fibers.

## Figures and Tables

**Table 1:** ^60^Co γ-Ray irradiation-initiated RAFT polymerization of AN under different conditions ^a^.

Entry	[AN]_0_:[CPDN]_0_	[AN]_0_ (mol/L)	Dose rate (kGy/h)	*M*_n,__th_^b^ (g/mol)	*M*_n,__NMR_ (g/mol)	*M*_n,__SEC_^c^ (g/mol)	*M*_w_/*M*_n_^c^	Conversion ^d^ (%)
1a	1000:1	5.09	1.8	22,500	8800	55,800	1.47	41.8
1b	500:1	5.09	1.8	7400	9000	22,000	1.28	27.0
1c	200:1	5.09	1.8	2000	2100	7000	1.12	16.0
2a	1000:1	5.09	1.2	17,200	11,400	44,200	1.38	31.9
2b	500:1	5.09	1.2	5800	7400	17,400	1.20	20.8
2c	200:1	5.09	1.2	1300	1400	5000	1.14	9.5
3a	1000:1	5.09	0.6	11,600	12,400	30,500	1.22	21.4
3b	500:1	5.09	0.6	3900	4300	12,700	1.15	13.6
3c	200:1	5.09	0.6	800	1000	4100	1.11	4.9
4a	500:1	10.18	1.8	2400	2700	9000	1.13	8.1

^a^ Reaction time = 5 h; ^b^ Under the conditions that the number of chains formed from the initiator can be neglected and the chain transfer agent is completely consumed, the theoretical molecular weight of PAN can be calculated by the following equation: *M*_n,th_ = $\frac{M_{\mathrm{MW}}\;\times \;[M]\;\times \;\mathrm{Conv}.\text{\%}}{\left[\mathrm{CTA}\right]}$ + CTA_MW_; ^c^ These molecular weights were calculated by polystyrene standards; ^d^ Conversion was calculated by the gravimetric method.

## Supplementary Materials

The following is available online at www.mdpi.com/2073-4360/9/1/26/s1. Figure S1: SEC chromatograms of 1a, 1b, 1c (Table 1); Figure S2: SEC chromatograms of 2a, 2b, 2c (Table 1); Figure S3: SEC chromatograms of PAN, DMF was used as the eluent and PS as the calibration standard (1.8 kGy/h; *t* = 5h; [AN]_0_ = 10.18 mol/L; [AN]_0_/[CPDN]_0_ = 1000/1); Figure S4: SEC chromatograms of polymerization of AN at 1.8 kGy/h ([AN]_0_/[CPDN]_0_ = 1000/1), DMF was used as the eluent and PS as the calibration standard.
