# Supplementary material for: A Green Platform for Preparation of the Well-Defined Polyacrylonitrile: 60Co γ-ray Irradiation-Initiated RAFT Polymerization at Room Temperature
Source: Polymers (Basel). 2017 Jan 17;9(1):26. doi: 10.3390/polym9010026 (PMC6432107; doi:10.3390/polym9010026)
Supplement: Supplementary file 1 [file polymers-09-00026-s001.pdf]

Supplementary Materials: A Green Platform for Preparation of the Well-Defined Polyacrylonitrile: ^{60}Co γ-ray-Irradiation-Initiated RAFT Polymerization at Room Temperature

Shuangshuang Zhang, Lu Yin, Junzhi Wang, Wei Zhang, Lifen Zhang and Xiulin Zhu

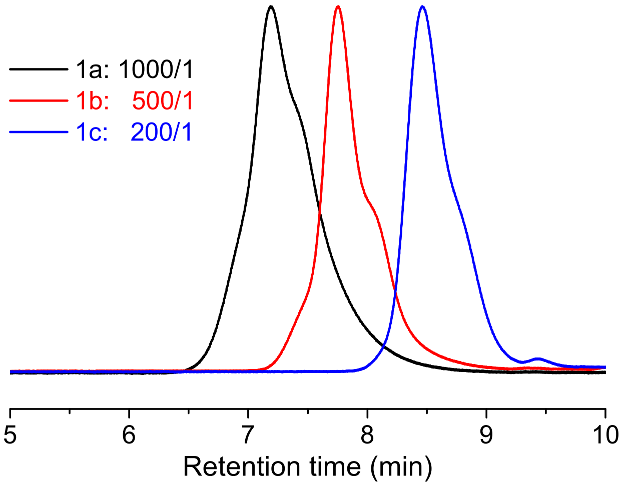

Figure S1. SEC chromatograms of 1a, 1b, 1c (Table 1). DMF was used as the eluent and PS as the calibration standard. $[AN]_0/[CPDN]_0$ = 1000/1, 500/1 or 200/1.

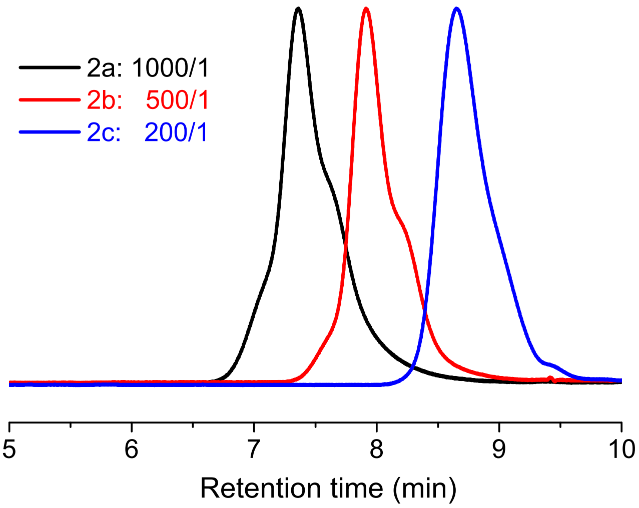

Figure S2. SEC chromatograms of 2a, 2b, 2c (Table 1). DMF was used as the eluent and PS as the calibration standard. $[AN]_0/[CPDN]_0$ = 1000/1, 500/1 or 200/1.

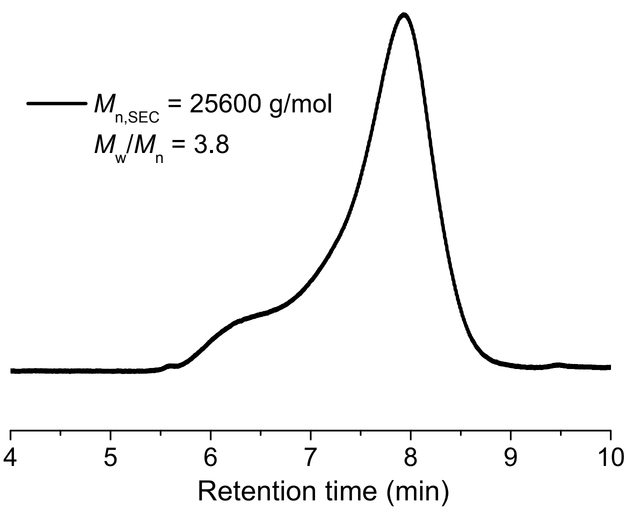

Figure S3. SEC chromatograms of PAN. DMF was used as the eluent and PS as the calibration standard. (1.8 kGy/h; t = 5 h; $[AN]_0$ = 10.18 mol/L; $[AN]_0/[CPDN]_0$ = 1000/1).

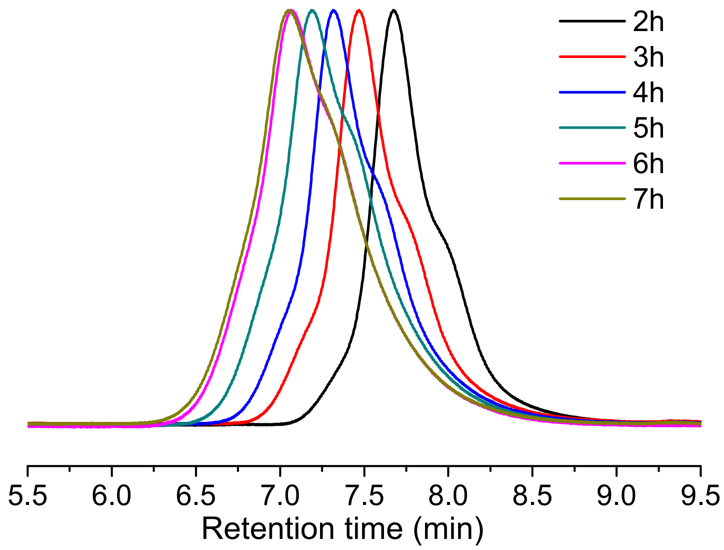

Figure S4. SEC chromatograms of polymerization of AN at 1.8 kGy/h ($[AN]_0/[CPDN]_0$ = 1000/1). DMF was used as the eluent and PS as the calibration standard.
